# Regulation of IL-33 by Oncostatin M in Mouse Lung Epithelial Cells

**DOI:** 10.1155/2016/9858374

**Published:** 2016-09-15

**Authors:** Carl D. Richards, Laura Izakelian, Anisha Dubey, Grace Zhang, Steven Wong, Karen Kwofie, Aatif Qureshi, Fernando Botelho

**Affiliations:** McMaster Immunology Research Centre, Department of Pathology and Molecular Medicine, McMaster University, Hamilton, ON, Canada

## Abstract

IL-33 modulates both innate and adaptive immune responses at tissue sites including lung and may play critical roles in inflammatory lung disease. Although IL-33 expression can be altered upon NF-Kappa B activation, here we examine regulation by Oncostatin M, a gp130 cytokine family member, in mouse lung tissue. Responses were assessed in BALB/c mouse lung at day 7 of transient overexpression using endotracheally administered adenovirus encoding OSM (AdOSM) or empty vector (AdDel70). Whole lung extracts showed induction of IL-33 mRNA (>20-fold) and protein (10-fold increase in immunoblots) by AdOSM relative to AdDel70. Immunohistochemistry for IL-33 indicated a marked induction of nuclear staining in alveolar epithelial cells* in vivo*. Oncostatin M stimulated IL-33 mRNA and IL-33 full length protein in C10 mouse type 2 alveolar epithelial cells in culture in time-dependent and dose-dependent fashion, whereas IL-6, LIF, IL-31, IL-4, or IL-13 did not, and TGF*β* repressed IL-33. IL-33 induction was associated with activation of STAT3, and pharmacological inhibition of STAT3 ameliorated IL-33 levels. These results indicate Oncostatin M as a potent inducer of IL-33 in mouse lung epithelial cells and suggest that an OSM/IL-33 axis may participate in innate immunity and inflammatory conditions in lung.

## 1. Introduction

Recent evidence supports important functions of the cytokine IL-33 in both innate and adaptive immune responses. IL-33 was first described as a nuclear factor from small endothelial venules [1, 2] and later by Schmitz et al. [3] as an extracellular ligand for ST2, which in turn had previously been described as an orphan receptor [4]. IL-33 showed ability to induce T helper 2 cytokines and subsequent work has further characterized IL-33 biology and roles in homeostasis and disease including bacterial and helminthic infection, allergy and chronic inflammation of lung, joint, skin, CNS, and cardiovascular tissues (as reviewed by others [5–8]). The extracellular soluble form of IL-33 interacts with its receptor complex composed of ST2 and IL-1RAP [9] and induces the NF-Kappa B and MAP kinase cell signaling pathways [3]. IL-33 is typically not secreted by living cells, where it is localized primarily to the nucleus, but it is released upon cell death/necrosis similar to other alarmins such as HMGB1 and IL-1*α* [5, 6]. Marked effects of excess soluble IL-33 in mice are indicated by lethal inflammation and autoimmunity in transgenic mice with high levels of soluble IL-33 bioavailability [10]. Less is known about the roles of IL-33 within the nucleus; however, cellular IL-33 is associated with heterochromatin [11] and can modulate NF-Kappa B signaling [12].

Elevated levels of IL-33 are found in mouse models of allergic airway disease and in chronic human lung conditions including severe asthma [5–8]. IL-33 influences Th2-skewed immune functions in allergic responses, where engagement of IL-33 receptor complexes on Th2 cells enhances Th2 responses. IL-33 induces eosinophil activation, exacerbates the OVA-induced airway inflammation model [13], and is required for maximal effects in models of HDM-induced airway inflammation [14]. However, IL-33 is also critical in innate immune responses such as LPS-induced systemic inflammatory responses and dextran-induced colitis with T cell-independent epithelial damage [15]. In addition, studies by Luzina et al. [16] indicate elevated expression of IL-33 in tissue from Idiopathic Pulmonary Fibrosis (IPF) patients, and other studies have implicated IL-33 in mouse models of lung fibrosis. Exogenous IL-33 increased severity in bleomycin-induced lung fibrosis [17], whereas inhibition of IL-33 pathways reduced lung fibrosis, as assessed by anti-IL-33 treatment in the bleomycin model or in ST2 KO mouse responses to bleomycin [18].

Human IL-33 was initially described in endothelium of lymph nodes [2] and subsequent work showed endothelial cell expression in many sites including chronically inflamed tissue. Constitutive IL-33 gene expression is evident in nuclei of endothelial and epithelial cells [11, 19] and mouse keratinocytes [20]. Myeloid cells can also express IL-33 upon induction by LPS/NF-Kappa B activation [21]. Among pulmonary tissue cells examined, airway smooth muscle cells [17, 22] and type 2 alveolar epithelial cells show IL-33 expression [7, 14]. Published studies on regulation of IL-33 by cytokines and growth factors indicate that TNF, IL-1, or GMCSF can induce IL-33 [14], whereas IFN-gamma appeared to repress IL-33 levels depending on the cell type [23]. IL-33 expression may be regulated in homeostasis and disease by other growth factors and cytokines. While the role of gp130 cytokines (including IL-6, LIF, Oncostatin M (OSM), IL-11, and IL-31) in regulation of IL-33 expression in lung cells is not yet known, we and others have previously shown that the gp130 cytokine OSM has homeostatic and proinflammatory activities and can modify extracellular matrix (ECM) [24–27]. Given the function of OSM in activating stromal cells and elevation of OSM levels in IPF and severe asthma [28, 29], putative regulation of IL-33 may be of importance in the modulation of innate immunity, chronic inflammation, and tissue repair in lung disease. In this study, we examined functions of Oncostatin M in modulating IL-33 expression in mouse lung* in vivo* and in mouse epithelial cells* in vitro*. OSM showed prominent regulation of IL-33 mRNA and full length IL-33 protein* in vivo* and induced cellular expression of full length IL-33 in mouse lung epithelial cells* in vitro* which was repressed with STAT3 inhibition. The results support a newly identified OSM-IL-33 axis in lung tissue cells and suggest an additional pathway in innate immune responses involving IL-33.

## 2. Materials and Methods

### 2.1. Cell Lines and Cytokines

Cell lines were cultured under standard conditions in medium containing 10% fetal bovine serum supplemented with 1% penicillin/streptomycin and 1% L-glutamine. The C10 type 2 alveolar epithelial cell line was cultured in RPMI as previously indicated [31] (gift from Dr. Chris Migliaccio, University of Montana). LA-4 cells were purchased from ATCC and cultured in DMEM/F12 media. Mouse recombinant cytokines including OSM, IL-6, LIF, IL-31, TNF*α*, TGF*β*, IL-4, IL-13, and IL-17A and mature IL-33 were purchased from R&D Systems (Minn., MN, USA). Pharmacological inhibitors were added at the following final concentrations prior to OSM stimulation: the STAT3 inhibitor Stattic at 5 *μ*M (30 min prior) (Abcam Biochemical, Toronto, Canada), the p38 inhibitor SB203580 at 5 *μ*M (1 hr prior) (Calbiochem, San Diego, CA), the ERK inhibitor PD98059 at 25 *μ*M (2 hr prior) (Calbiochem), and the AKT inhibitor AKTX at 5 *μ*M (1 hr prior) (Calbiochem).

### 2.2. Adenoviral Vector* In Vivo* Experiments

Adenovirus encoding mouse OSM (AdOSM) or empty virus (AdDel70) has been previously described and was used to transiently express virus encoded genes in lungs by endotracheal administration as previously indicated [30, 31]. At day 7 after administration of vectors, each at 5 × 10^7^ pfu with *n* = 5 mice per treatment, the mice were culled, bronchoalveolar lavage was completed, and the right lung lobes were extracted and snap-frozen. Portions were later processed either for RNA analysis or for protein analysis as previously described [32]. The left lung lobe was excised, inflated/fixed with formaldehyde, and subsequently processed for histology and immunohistochemistry.

### 2.3. Immunohistochemistry (IHC) and Quantification

IHC for IL-33 was completed similarly to that as previously described [14] and as follows: paraffin embedded sections of left lungs of mice treated with Ad vectors as indicated were deparaffinized, hydrated sequentially with absolute, 95%, and 70% alcohol, and then washed in 3% hydrogen peroxide (to block endogenous peroxide) and then in TBS/tween/0.1% saponin. The sections were then blocked with horse serum as part of the ImmPRESS system (Vector Labs) in 0.1% saponin, washed (TBS/tween/0.1% saponin), and then incubated with goat anti-mouse IL-33 primary antibody (cat# AF3626, R&D Systems) at varying concentrations (2.5 *μ*g/mL, 1.2 *μ*g/mL, and 0.6 *μ*g/mL) for 1 hr. After washing, ImmPRESS secondary Ab (horse anti-goat IgG) was added for 30 min and then washed and antigen detected with DAB reagent (Dako). For prosurfactant protein C (SPC), methods were as described previously [14] using 1 : 2000 anti-SPC polyclonal Ab (Millipore) and Envision + rabbit (Dako). For CD31 stain, antigen retrieval was used (Dako target retrieval) before rat anti-mouse CD31 (1 : 50 dilution, overnight rt) and biotinylated rabbit anti-rat (mouse absorbed) at 1 : 100 (Vector Labs) and then revealed with Envision-plus-rabbit-TRU (Dako K4003). Mayer's hematoxylin was used for counterstain. Slides processed through the procedures without primary antibody were negative for any staining. For quantification, IL-33^+^ cells were enumerated per high power field (400x) with 5 fields counted per left lung from at least 3 separate mice per Ad vector treatment.

### 2.4. Analysis of Protein (Immunoblot or ELISA) and RNA Levels

15–20 *μ*g of protein was loaded on 10% or 12% SDS-PAGE gels and following separation proteins were transferred to a nitrocellulose membrane by standard methods [31] and blocked for one hour at room temperature using LI-COR Odyssey blocking buffer (Mandel). BLUeye molecular weight protein ladder (FroggaBio, Toronto, Canada) was run on all protein gels. Blots were then probed using the following antibodies at 4°C overnight: actin I-19 and phospho-p38 (Santa Cruz Biotechnology), diluted 1 : 2000; mouse IL-33 (R&D Systems), diluted 1 : 1000; phosphorylated STAT3 (Cell Signaling Technology), diluted 1 : 2000; total STAT3 (Cell Signaling Technology), diluted 1 : 1000; and SMAD1, diluted 1 : 1000 (Cell Signaling Technology). Phospho-SMAD1/5/8 (1 : 1000 dilution) was purchased from Millipore. Primary antibodies were detected using LI-COR anti-rabbit or anti-goat IRDye infrared secondary antibodies at 1 : 5000 or 1 : 10000 dilution, respectively, and imaged using the LI-COR Odyssey infrared scanner. Blots were stripped using LI-COR NewBlot Nitro Stripping Buffer, as per manufacturer's instructions, and then blocked and reprobed for different proteins. Densitometry analysis of immunoblot band intensity was performed using Image Studio Lite software (LI-COR). IL-33 protein bands were normalized to actin; pSTAT3 was normalized to total STAT3. Cell lysates were compared relative to untreated control samples to quantify fold changes in signal, and lung homogenate samples were compared relative to lung homogenate samples from mice treated with AdDel70. For assessing soluble IL-33 in cell culture supernatants, DuoSet antibody pairs for mouse IL-33 were purchased from R&D Systems Inc. and used as per manufacturer's protocols. The limit of detection for the assay was 15 pg/mL. In assessing mRNA levels, RNA was prepared following manufacturer's protocol using PureLink RNA Mini kits and analyzed by real-time quantitative PCR. Predetermined assay reagents (PDAR probes) were purchased for IL-33 and ST2/IL1RL1 (Thermo Fisher).

### 2.5. Statistical Analysis

GraphPad Prism version 5.0 was used to generate graphs to display the results and for statistical analysis. Figures represent mean ± standard error of measurement (SEM). The one-way analysis of variance (ANOVA) test was used to evaluate statistical differences using Tukey's* post hoc* multiple comparisons test at a significance level of 0.05. *P* < 0.05 was considered statistically significant.

## 3. Results

**Figure 1:**
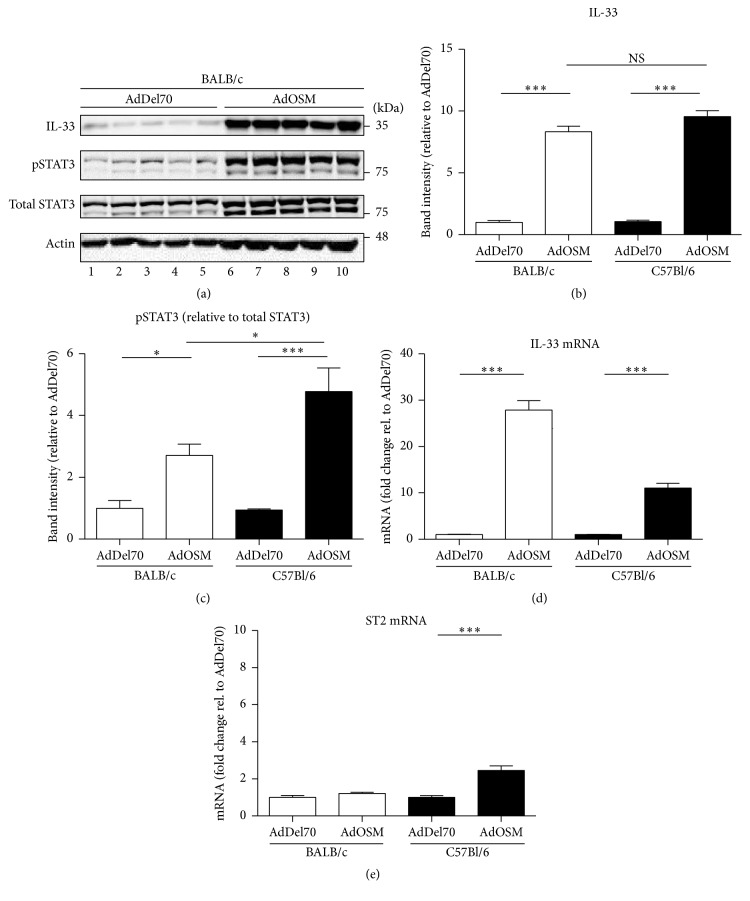
AdOSM induces IL-33 expression* in vivo*. Mice were culled after 7 days of an endotracheal administration of AdDel70 or AdOSM vectors (*n* = 5 for each treatment) and lung tissue was prepared for protein or RNA analysis as described in Materials and Methods. (a) Lung homogenate protein samples of BALB/c mice (lanes 1–5: individual mice treated with AdDel70; lanes 6–10: individual mice treated with AdOSM) were probed for IL-33, pSTAT3, total STAT3, and actin as indicated. (b, c) Quantification of band intensity was completed as described in Materials and Methods and white bars represent samples from BALB/c mice and black bars represent samples from C57BL/6 mice treated in parallel. IL-33 levels were normalized to actin; pSTAT3 was normalized to total STAT3. (d, e) RNA from total lung was probed by qRT-PCR for IL-33 or the IL-33 receptor chain ST2. The results represent the mean ± SEM, and statistical significance is indicated, where *P* < 0.05 (*∗*) or *P* < 0.001 (*∗∗∗*). These data are representative of at least two experiments with identical trends. BLUeye molecular weight marker migration is shown on the right of panel (a).

We have previously shown that transient overexpression of OSM using Ad vector expressing mouse OSM (AdOSM) induces inflammatory responses and increases extracellular matrix in lungs of mice [30, 31]. To determine whether IL-33 is expressed and/or regulated in this system, we examined both protein and mRNA levels in mice treated with control AdDel70 vector or AdOSM vector (Figure 1). The AdDel70 vector is without the mOSM cDNA insert, but otherwise it has the identical backbone and is used as the control vector as previously indicated [30, 31]. Immunoblots of total lung protein extracts showed consistent elevation of IL-33 signal by AdOSM and this correlated with pSTAT3 activation (Figure 1(a)). AdDel70 alone did not upregulate IL-33 at day 7, as compared to naïve uninfected mice (data not shown). Quantification of IL-33 (corrected to actin) and pSTAT3 (corrected to total STAT3) showed significant induction of IL-33 levels in both BALB/c (Figures 1(a)–1(c)) and C57BL/6 (Figures 1(b) and 1(c) and supplemental Figure  1 in Supplementary Material available online at http://dx.doi.org/10.1155/2016/9858374) total lung extracts. The antibody detected prominent bands corresponding to full length IL-33 (migrated at approximately 35 kD), whereas mature IL-33 signal (recombinant mature IL-33 migrated at approximately 18-19 kD) was absent/low in mouse lung extracts of both AdDel70- and AdOSM-treated BALB/c mice (supplemental Figure  2). To assess expression of IL-33 and IL-33 receptor chain (IL-33R/ST2) mRNA levels, total lung homogenates were examined by qRT-PCR (Figures 1(d) and 1(e)). The data showed induction of IL-33 mRNA in both BALB/c (>20-fold) and C57BL/6 (>10-fold) mouse strains. In contrast, mRNA levels of ST2 were not altered in BALB/c mice and minimally elevated (2-fold) in C57BL/6 mice at the time point examined.

**Figure 2:**
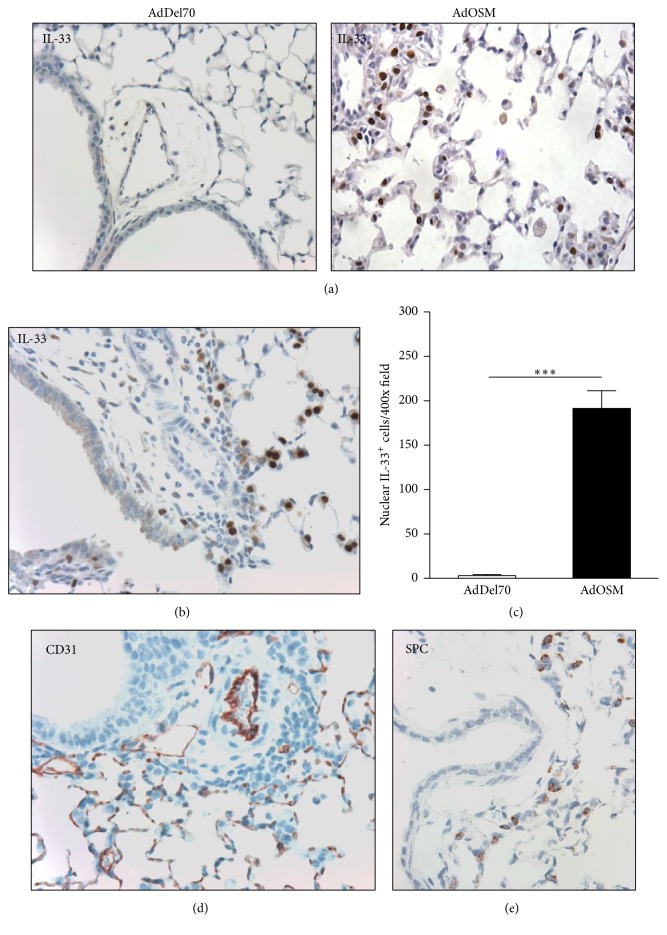
IL-33 is expressed in alveolar type II cells. BALB/c mice were culled 7 days after endotracheal administration of AdDel70 or AdOSM vector and left lungs analyzed by specific immunohistochemistry for IL-33 as described in Materials and Methods. (a) Representative micrographs of lungs from AdDel70 or AdOSM as indicated with IL-33 as brown-red signal. (c) Quantification of nuclear IL-33^+^ epithelial cells per 400x field (*n* = 3 mice/group) in lung sections of mice given AdDel70 or AdOSM vector (day 7). Lung sections at day 7 from BALB/c mice given AdOSM were stained for (b) IL-33, (d) CD31, and (e) prosurfactant protein C (SPC). And statistical significance is indicated, where *P* < 0.001 (*∗∗∗*).

To determine which cells in the lung express IL-33 upon transient OSM overexpression, we examined lung sections by specific IL-33 immunohistochemistry.  Figure 2(a) showed some IL-33 staining in AdDel70-treated BALB/c mice which was limited to the nucleus of IL-33-positive cells throughout the parenchyma. In contrast, AdOSM-treated mice showed lungs with marked increases in number and intensity of IL-33-stained cells, localized predominantly to the nucleus of cells with morphology of type 2 alveolar epithelial cells (Figures 2(a) and 2(b)). Quantification of IL-33-nuclear positive cells per 400x magnification is shown in Figure 2(c) and indicated significant increases in numbers of cells. We also stained for prosurfactant protein C (SPC) to identify type 2 alveolar epithelial cells (Figure 2(e)). A similar distribution of IL-33 and SPC staining was observed, consistent with expression of IL-33 by type 2 alveolar epithelial cells, as shown previously in other systems of mouse lung inflammation [14]. Since it has been shown that endothelial cells express IL-33 in other systems, we examined mouse lung sections stained for CD31, a typical vascular endothelial cell marker. CD31 staining was evident on the luminal surface of blood vessels; however, these cells were not positive for IL-33 in serial sections (Figure 2(d)). To confirm that AdOSM induced the same effects in BALB/c mouse lung as previously described in this system [31], inflammatory cell accumulation in bronchoalveolar lavage (BAL) fluid and histopathology of lung tissue sections are shown in supplementary Figure  4. The data indicated that mice showed the expected elevation of cells in BAL as well as increases in epithelial cell hyperplasia, thicker alveolar walls, and inflammatory cell accumulation in tissue sections, in response to AdOSM.

**Figure 3:**
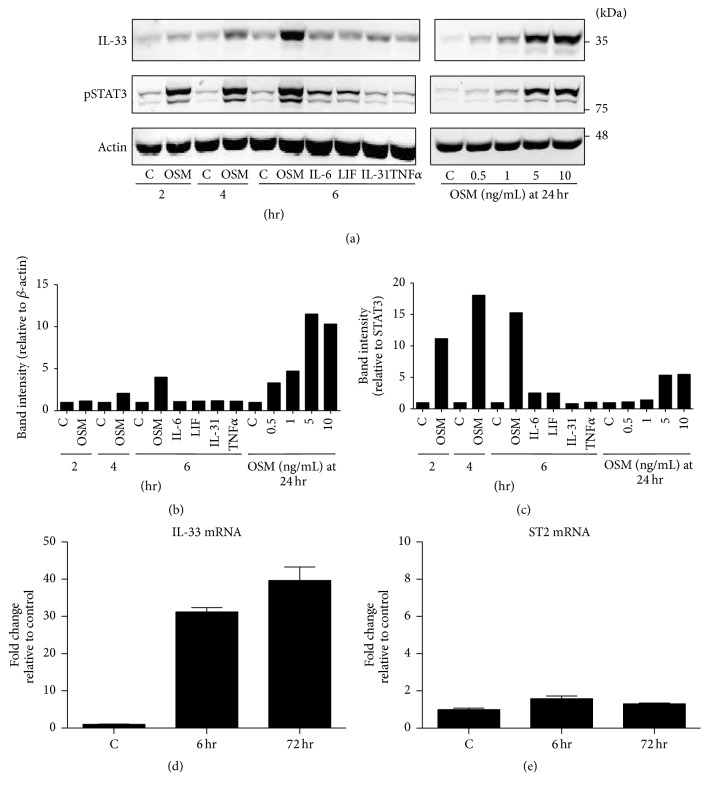
C10 lung epithelial cell IL-33 responses to OSM* in vitro. *C10 alveolar epithelial cells were stimulated with the indicated cytokines (5 ng/mL) and lysed after 2, 4, and 6 hours. C10s were also stimulated with varying concentrations of recombinant OSM and lysed after 24 hours. (a) Cell lysates were probed for IL-33, pSTAT3, and actin protein as indicated. (b, c) Quantification of bands was completed using Image Studio Lite, where IL-33 was corrected for actin and pSTAT3 was corrected for total STAT3. (d, e) IL-33 and ST2 mRNA expression in C10 cells stimulated with or without 5 ng/mL OSM for the indicated times as assessed by qRT-PCR (C: control/unstimulated). The results represent the mean ± SEM. BLUeye molecular weight marker migration is shown on the right of panel (a).

Since the data indicated type 2 epithelial cells were strongly responsive to AdOSM* in vivo*, we further explored expression of IL-33* in vitro*. We first tested whether OSM could directly regulate IL-33 in mouse type 2 alveolar epithelial cells using the C10 cell line derived from BALB/c [31]. C10 cells stimulated with OSM resulted in time-dependent increases (evident at 4 and 6 hours and more prominently at 24 hours) and dose-dependent increases (evident with 0.5 ng/mL OSM stimulus and markedly with 5 ng/mL OSM at 24 hr) in IL-33 signal detected by immunoblots of C10 cell lysates (Figures 3(a) and 3(b)). We could not detect presence of mature IL-33 (approximately 18-19 kD bands, as indicated by inclusion of recombinant mature IL-33 in supplemental Figure  2) in unstimulated or OSM-stimulated C10 cell lysates. Similar results were observed using a monoclonal anti-IL-33 antibody (Nessy-1 antibody from Abcam, data not shown).  Figure 3(b) shows a quantitative 2-fold, 4-fold, and 12-fold increase in IL-33 signal at 4 hr, 6 hr, and 24 hr, respectively, upon stimulation with 5 ng/mL OSM (Figure 3(b)). Other gp130 cytokines including IL-6, LIF, or IL-31 did not stimulate IL-33 detectably in C10 cell lysates in comparison to OSM (Figures 3(a) and 3(b)) nor did TNF*α*. Activation of STAT3, as assessed by pSTAT3 immunoblots, was evident in lysates of OSM-stimulated cells in dose-dependent fashion, whereas IL-6, LIF, IL-31, or TNF did not elevate pSTAT3 (Figures 3(a) and 3(c)). When supernatants of cells from any of these conditions were tested for IL-33 by specific ELISA, no detectable soluble IL-33 was evident (not shown) at the level of sensitivity of the ELISA (15 pg/mL, R&D systems). IHC staining for IL-33 in C10 cell cultures* in vitro* showed detectable IL-33 in nuclei in unstimulated cells and a marked increase in nuclear staining upon OSM stimulation (supplemental Figure  3) consistent with the IL-33 staining in whole lung (Figure 2). To determine whether OSM regulated IL-33 or ST2 expression at the mRNA level* in vitro*, C10 cellular extracts were assessed by qRT-PCR and showed approximately 30-fold and 40-fold increases in IL-33 mRNA at 6 hours or at 72 hours, respectively, after OSM stimulation (Figure 3(d)), whereas the IL-33 receptor chain ST2 mRNA levels did not change (Figure 3(e)).

**Figure 4:**
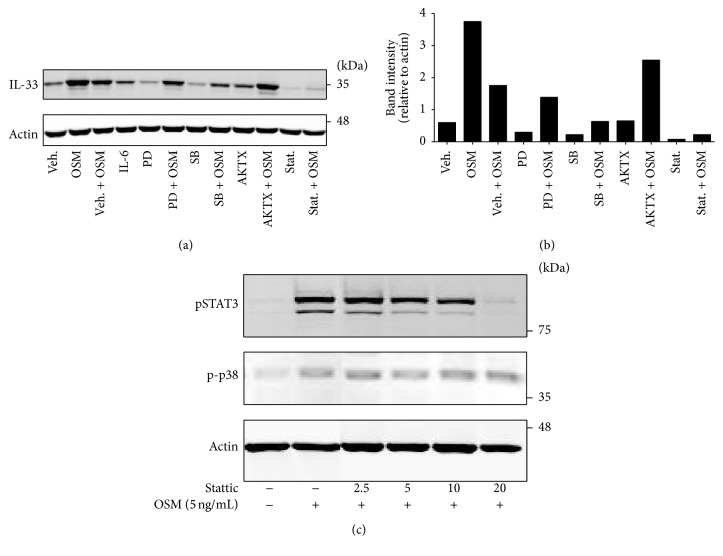
STAT inhibition reduces IL-33 induction* in vitro*. (a) C10 alveolar epithelial cells were treated with the indicated inhibitors prior to stimulation with recombinant OSM (5 ng/mL) and lysed at 6 hours (Veh.: vehicle; PD: PD98059; SB: SB203580; AKTX: AKT inhibitor; Stat.: Stattic). Cell lysates were probed for IL-33 protein. (b) Immunoblot band intensities for IL-33 were quantified and corrected for actin as in Materials and Methods. (c) C10 cells were inhibited with varying concentrations of Stattic for 30 minutes prior to stimulation with OSM. Cells were lysed after 20 minutes, and lysates were probed for pSTAT3, phospho-p38, and actin, as indicated. BLUeye molecular weight marker migration is shown on the right of panels (a) and (c).

Since induction of IL-33 protein levels was associated with activation of STAT3 both* in vivo* and* in vitro* (Figures 1 and 3), we then assessed whether pharmacological inhibition of STAT3 affected IL-33 induction in C10 cells. In Figure 4(a) (immunoblots) and Figure 4(b) (quantitative densitometry), data showed that preincubation of cells with the STAT3 inhibitor Stattic, or the p38 inhibitor SB203580, reduced the signal of IL-33 compared to vehicle alone at 6 hr of OSM stimulation. As compared to vehicle alone, the ERK inhibitor PD908059, and the Akt inhibitor AKTX, did not modulate OSM induction of IL-33 signal. Stattic inhibited early OSM-induced pSTAT3 levels (20-minute time point) in dose-dependent fashion but did not affect the OSM-induced levels of phospho-p38 (Figure 4(c)). Absence of pSTAT3 activation corresponded to reduced levels of IL-33 protein expression (Figure 4(a)). This supports a role of STAT3 activation in the regulation of IL-33 levels by OSM in C10 cells.

**Figure 5:**
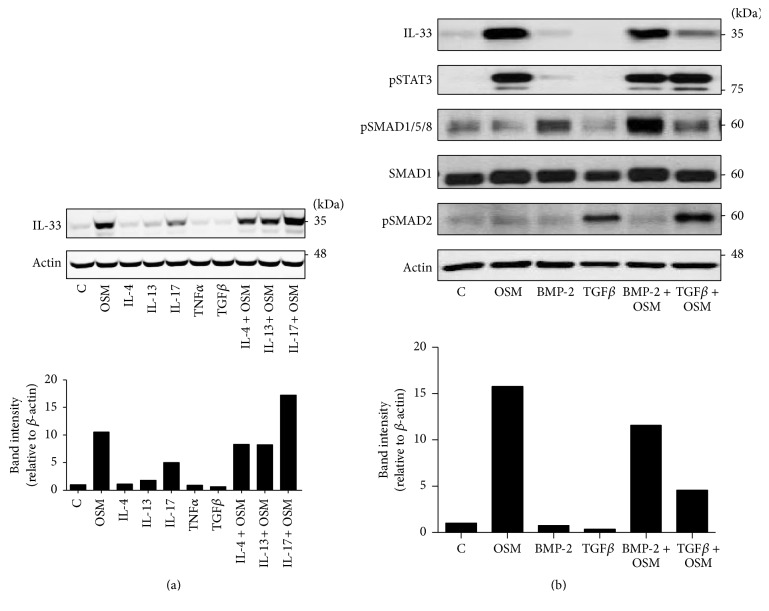
Cytokine modulation of OSM-induced IL-33* in vitro*. C10 alveolar epithelial cells were stimulated with the indicated cytokines (5 ng/mL) and lysed after 24 hours. (a) Cell lysates were probed for IL-33 and actin as indicated (upper panel) and quantified as previously indicated (lower panel). (b) IL-33, pSTAT3, SMAD proteins, and actin protein expression levels were assessed by immunoblots of lysates from C10 cells stimulated with the indicated cytokines (all at 5 ng/mL). IL-33 band intensity was quantified as described in Materials and Methods (lower panel of (b)). BLUeye molecular weight marker migration is shown on the right of panels (a) and (b).

**Figure 6:**
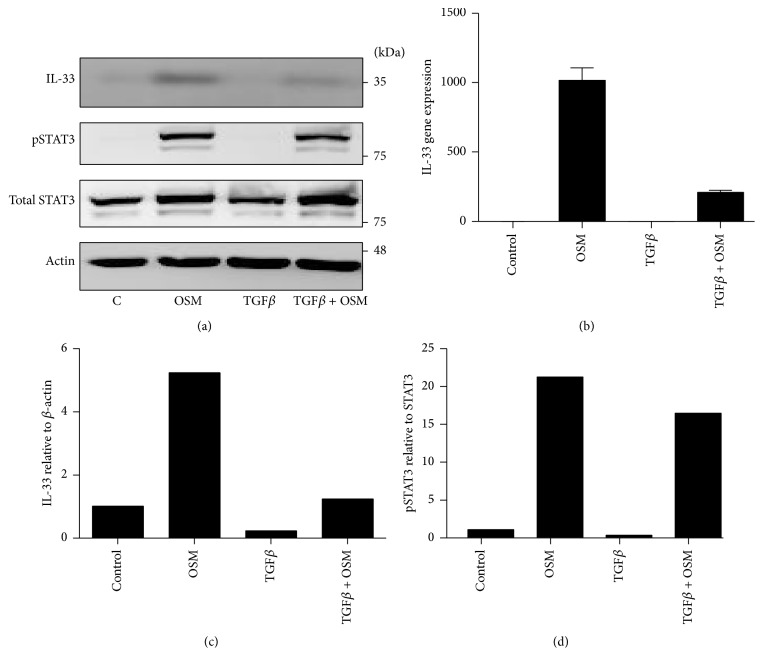
(a) LA-4 cell lysates were probed for IL-33, pSTAT3, total STAT3, and actin as indicated. (b) Quantification of IL-33 band intensity. (c) mRNAs from LA-4 cells were extracted and probed by quantitative PCR for IL-33 gene expression. Results represent the mean ± SEM. (d) Quantification of phospho-STAT3 band intensity relative to total STAT3 protein. BLUeye molecular weight marker migration is shown on the right of panel (a).

We then compared OSM induction of IL-33 to the activity of Th2 cytokines IL-4 and IL-13, as well as the Th17 cytokine IL-17A, in their ability to regulate IL-33 in C10 cell cultures.  Figure 5(a) shows that OSM and to a lesser extent IL-17A stimulation elevated IL-33 signal, whereas IL-4, IL-13, and TGF*β* alone did not elevate IL-33. In combination, IL-4 or IL-13 did not detectably affect OSM-induced IL-33, whereas IL-17A and OSM combination resulted in higher levels of IL-33 signal compared to either cytokine alone. IL-33 has been implicated in exacerbation of ECM accumulation in mouse lungs as shown by others [17, 18]. Since OSM, BMP-2, and TGF*β* are cytokines that can induce ECM in various systems, we tested each in their ability to regulate IL-33 in the mouse epithelial C10 cells (Figure 5(b))* in vitro*. Results indicate that stimulation by OSM, but not BMP-2 or TGF*β*, increased IL-33 cellular protein in C10 cell lysates. BMP-2 activated its canonical signaling pathway as indicated by elevated pSMAD1/5/8, and TGF*β* activated its canonical pathway as indicated by elevated pSMAD2 in the same C10 cell lysates. This indicates that, despite functional receptors and signaling in these cells, neither TGF*β* nor BMP2 alone could induce IL-33 expression. When the cytokines were added in combination, TGF*β* inhibited IL-33 induction by OSM in C10 cell cultures. To determine whether similar OSM responses were also evident in a second mouse epithelial cell line, we assessed mouse LA-4 in similar fashion. Although the level of IL-33 signal was generally lower in LA-4 cells than in C10 cells, Figure 6(a) shows that OSM induced IL-33 protein signal in LA-4 lysates, whereas TGF*β* alone did not, and TGF*β* again reduced IL-33 signal when added in combination with OSM. Assessment of mRNA levels of IL-33 in LA-4 cells (Figure 6(b)) showed results consistent with the protein analysis, where OSM induced IL-33 mRNA levels, TGF*β* alone did not, and TGF*β* reduced IL-33 mRNA when added in combination with OSM.

## 4. Discussion

Our results show that transient gene expression of OSM in lungs of mice induces IL-33 mRNA and protein levels in whole lung extracts* in vivo*. Alveolar type 2 cells but not vascular endothelial cells expressed markedly elevated IL-33* in vivo*, and OSM directly induced IL-33 in mouse alveolar epithelial type 2 C10 cells* in vitro*. IL-33 induction was associated with STAT3 activation both* in vivo* and* in vitro*. This is the first identification of OSM as a participant in IL-33 regulation in lung and suggests additional mechanisms of IL-33 regulation in pulmonary inflammation and pathology.

The results of IHC analysis of lung sections and C10 epithelial cells* in vitro* indicated that type 2 alveolar cells were prominent responders to OSM in IL-33 expression. Other stromal cells such as fibroblasts, endothelial cells, and smooth muscle cells have been shown to express IL-33 in other systems and may also contribute to IL-33 levels in total lung, although here we focused on alveolar type 2 cells due to their robust response in this system. Others have also shown IL-33 induction in alveolar type 2 cells in lung inflammatory models including the HDM model of allergic airway inflammation in BALB/c mice [14] and in lung alveoli upon papain-induced allergic airway inflammation in C57BL/6 mice. Recent work has also shown that systemic expression of OSM in mice, using the same AdOSM vector used here but administered intramuscularly, induced IL-33 expression in liver endothelial cells* in vivo* [33]. Whether lung tissue IL-33 expression can be modified by systemic OSM elevation is not yet known. In the present work we could not detect IL-33 upregulation in lung endothelial cells with local pulmonary OSM overexpression (Figure 2). Pichery et al. [20] have recently shown that IL-33 is not constitutively expressed in mouse lung endothelium (IL-33 immunostaining) but is expressed to some extent in simple cuboidal cells in lung epithelium of C57BL/6 mice. Our studies indicated low/no staining of cuboidal epithelial cells in BALB/c mice by IHC, and this was minimally altered upon overexpression of OSM (Figure 2). This may reflect technical differences in detection systems used or possibly differences in BALB/c and C57BL/6 mouse strains in cell expression of IL-33 protein. Moreover, recent work by Sundnes et al. [34] has also suggested significant species differences in IL-33 expression between mouse and human cells/tissues.

We observed induction of IL-33 protein levels by OSM but not by IL-6, LIF, or IL-31 (Figure 3)* in vitro* and this was consistent with relative activation of STAT3 by each of these cytokines in the same cell lysates. OSM induces a number of signaling pathways in connective tissue cells including STATs 1 and 3, MAPK PI3K, and PKC delta, depending on the cell systems examined [26, 27]. Pronounced and prolonged STAT3 activation is characteristic of OSM responses both* in vitro* and* in vivo* [31, 35]. In the systems examined here, pharmacological inhibition of STAT3 activation resulted in repressed IL-33 induction. Other works have shown that OSM can elevate IL-33 in osteoblast cell cultures and may be involved in the anabolic activity of OSM in bone formation [36] in musculoskeletal tissue; however, the involvement of STAT3 was not explored. A sequence search of the murine IL-33 promoter in the NCBI nucleotide database reveals multiple STAT binding sites, suggesting that STAT3 activation can directly regulate transcription of the IL-33 gene, although confirmation of this mechanism requires further study.

Upon testing Th2 cytokines (IL-4 and IL-13) or IL-17A, IL-17A enhanced IL-33 alone and additively when used in combination with OSM to stimulate C10 cells. On the other hand, IL-4 or IL-13 had no detectable activity. Whether similar effects are evident in other lung cell types remains to be established, and regulations by OSM and IL-17A in lung fibroblasts or lung smooth muscle cells are currently being investigated to determine whether OSM/IL-17A regulation of IL-33 is a more general effect. Conversely, TGF*β* ameliorated IL-33 levels in C10 as well as LA-4 alveolar epithelial cells* in vitro* (Figures 5 and 6). Although the mechanism by which TGF*β* suppresses IL-33 protein levels in lung cells is unclear (we did not observe modulation of OSM-induced STAT3 signals), suppression of IL-33 expression could indicate another function of TGF*β* in modulating functions in immunity. A previous study showed TGF*β* inhibition resulted in increased IL-33^+^ macrophages and increased pathology in colon of DSS treated mice [37]; however, whether stromal cell expression of IL-33 was modulated in that study was not indicated. Collectively, our results indicate that OSM action on IL-33 is not shared by Th2 cytokines or other gp130 cytokines in mouse lung epithelial cells. Others have shown that OSM potentiated responses to IL-4 and IL-13 in regulation of chemokines such as eotaxin-1 in other cell types [32]. However, our results here indicate that the regulation of IL-33 appears to be independent of IL-4/IL-13 and STAT6 signaling in mouse lung epithelial cells.

IL-33 is expressed as a full length protein (migrates at approximately 35 kD in immunoblots) which can be cleaved to yield smaller isoforms. Extracellular full length IL-33 is bioactive at its receptor complex as are mature forms cleaved by elastase and cathepsin G which yield smaller and more active mature IL-33 [38]. IL-33 can also be processed during apoptosis by caspase 3 and 8 to yield cleaved IL-33; however, such isoforms are not biologically active [39]. Immunoblots for IL-33 enabled us to simultaneously assess isoforms of IL-33 present as well as signaling molecules in cell/tissue extracts. The data indicate that, in cell lysates of C10 cells, and in whole lung homogenates of mice treated* in vivo*, full length IL-33 is the major form elevated due to OSM. We could not detect IL-33 species in lung homogenates or C10 cell samples by Western blot which corresponded to the size of recombinant bioactive mature 18-19 kD form of IL-33 (supplemental Figure  2). Upon prolonged exposure of detection by immunoblots, very minor bands lower than 35 kD were observed in total lung extracts (supplemental Figure  2); however, it is not clear if these are cleaved forms of IL-33 or nonspecific protein species. Such bands were not evident in cell culture lysates, and clearly the full length form is the major IL-33 species induced in this system. We could not detect increases in soluble IL-33 in bronchoalveolar lavage (BAL) of the AdOSM- or AdDel70-treated BALB/c mice or in supernatants of C10 type 2 alveolar epithelial cells as measured by commercially available ELISA (not shown). Thus, in this system using BALB/c mice* in vivo* and epithelial cells derived from BALB/c mice* in vitro*, mature IL-33 was not detectable. Previous work by Luzina et al. [16] has shown that overexpression of mature IL-33 using adenovirus vectors in C57BL/6 mouse lung mediates ST2-dependent eosinophil accumulation, goblet cell increases, and higher IL-4, IL-5, and IL-13 levels, whereas Ad vector encoding full length IL-33 did not [16]. Our previous results show that BALB/c mice treated with AdOSM do not show increased levels of eosinophil accumulation or Th2 cytokines (IL-4 and IL-13) in BAL [31], consistent with a lack of soluble full length or mature active IL-33 presence and/or activity in BALB/c mice in our system. On the other hand, we and others show that OSM induces Th2 cytokine skewing and pulmonary eosinophilia in C57BL/6 mice [29, 31]. It is possible that such effects in C57BL/6 mice are mediated in part by elevated soluble IL-33 in the C57BL/6 mouse strain, but this needs further study.

In addition to functions upon release and action at the ST2/IL-1RAP receptor complex, IL-33 has been shown to have roles as a nuclear factor that can bind chromatin and regulate NF-kB function [11, 12]. The increase of nuclear IL-33 upon OSM stimulation may thus contribute to gene regulation, and future work using IL-33 deficient cells (either gene knockout or siRNA knockdown) could assess this function. Our results also lead us to suggest that OSM presence in combination with necrotic cell death/damage will lead to increased release of extracellular IL-33. Others have shown that influenza virus infection of mouse lung results in increased levels of soluble IL-33 [40]. Kearley et al. [41] have recently shown that infection of mouse epithelial TC1 cells with influenza A or RSV induces release of immunodetectable IL-33. In chronic lung conditions, where OSM is elevated (such as IPF and severe asthma) [28, 29], IL-33 is also upregulated [17, 22]. We postulate that elevated OSM enhances the cellular pool of IL-33, which in combination with epithelial cell damage/death results in increased release of soluble IL-33 and its subsequent actions as an alarmin. Collectively, our results suggest an OSM-IL-33 axis exists which engages stromal cells and contributes to innate and inflammatory mechanisms of disease. The enhancement of innate immune functions that release IL-33 may also influence ongoing and/or subsequent adaptive immune responses.

## Supplementary Material

Supplemental Figure 1 demonstrates an induction of full-length IL-33 protein (35 kDa) and phospho-STAT3 (79, 86 kDa) by Western blot, in whole lung homogenates of C57Bl/6 mice treated with AdOSM, in comparison to AdDel70 control vector. Samples were also probed for β-Actin and total-STAT3 as loading controls.
Supplemental Figure 2 shows that the antibody in Western blots detected prominent bands corresponding to full length IL-33 (approximately 35kDa) induced by OSM whereas mature IL-33 signal (recombinant mature IL-33 at approximately 18-19 kDa) was absent/low in mouse lung extracts of both AdDel70 and AdOSM-treated BALB/c mice, as well as C10 cell culture lysates stimulated with OSM. Recombinant mature IL-33 (R&D) was run on the same gel for comparison and samples were probed for *β*-Actin as a loading control.
Supplemental Figure 3 contains results from IHC staining for IL-33 in C10 cell cultures in vitro. The data showed detectable IL-33 in nuclei of un-stimulated cells and a marked increase in nuclear staining upon OSM stimulation.
Supplemental Figure 4 confirms that AdOSM treatment resulted in the same effects in BALB/c mouse lung as previously described in this system [31], including inflammatory cell accumulation in broncho-alveolar lavage (BAL) fluid and histopathology of lung tissue sections. The results indicated expected elevation of cells in the BAL fluid as well as increases in epithelial cell hyperplasia, thicker alveolar walls, and inflammatory cell accumulation in tissue sections, in response to AdOSM.

## References

[1] Onda H., Kasuya H., Takakura K. (1999). Identification of genes differentially expressed in canine vasospastic cerebral arteries after subarachnoid hemorrhage. *Journal of Cerebral Blood Flow and Metabolism*.

[2] Baekkevold E. S., Roussigné M., Yamanaka T. (2003). Molecular characterization of NF-HEV, a nuclear factor preferentially expressed in human high endothelial venules. *American Journal of Pathology*.

[3] Schmitz J., Owyang A., Oldham E. (2005). IL-33, an interleukin-1-like cytokine that signals via the IL-1 receptor-related protein ST2 and induces T helper type 2-associated cytokines. *Immunity*.

[4] Tominaga S.-I. (1989). A putative protein of a growth specific cDNA from BALB/C-3T3 cells is highly similar to the extracellular portion of mouse interleukin 1 receptor. *FEBS Letters*.

[5] Liew F. Y., Pitman N. I., McInnes I. B. (2010). Disease-associated functions of IL-33: the new kid in the IL-1 family. *Nature Reviews Immunology*.

[6] Haraldsen G., Balogh J., Pollheimer J., Sponheim J., Küchler A. M. (2009). Interleukin-33 - cytokine of dual function or novel alarmin?. *Trends in Immunology*.

[7] Molofsky A. B., Savage A. K., Locksley R. M. (2015). Interleukin-33 in tissue homeostasis, injury, and inflammation. *Immunity*.

[8] Miller A. M. (2011). Role of IL-33 in inflammation and disease. *Journal of Inflammation*.

[9] Chackerian A. A., Oldham E. R., Murphy E. E., Schmitz J., Pflanz S., Kastelein R. A. (2007). IL-1 receptor accessory protein and ST2 comprise the IL-33 receptor complex. *Journal of Immunology*.

[10] Bessa J., Meyer C. A., de Vera Mudry M. C. (2015). Altered subcellular localization of IL-33 leads to non-resolving lethal inflammation. *Journal of Autoimmunity*.

[11] Carriere V., Roussel L., Ortega N. (2007). IL-33, the IL-1-like cytokine ligand for ST2 receptor, is a chromatin-associated nuclear factor in vivo. *Proceedings of the National Academy of Sciences of the United States of America*.

[12] Ali S., Mohs A., Thomas M. (2011). The dual function cytokine IL-33 interacts with the transcription factor NF-*κ*B to dampen NF-*κ*B-stimulated gene transcription. *Journal of Immunology*.

[13] Stolarski B., Kurowska-Stolarska M., Kewin P., Xu D., Liew F. Y. (2010). IL-33 exacerbates eosinophil-mediated airway inflammation. *The Journal of Immunology*.

[14] Llop-Guevara A., Chu D. K., Walker T. D. (2014). A GM-CSF/IL-33 pathway facilitates allergic airway responses to sub-threshold house dust mite exposure. *PLoS ONE*.

[15] Oboki K., Ohno T., Kajiwara N. (2010). IL-33 is a crucial amplifier of innate rather than acquired immunity. *Proceedings of the National Academy of Sciences of the United States of America*.

[16] Luzina I. G., Pickering E. M., Kopach P. (2012). Full-length IL-33 promotes inflammation but not Th2 response in vivo in an ST2-independent fashion. *The Journal of Immunology*.

[17] Luzina I. G., Kopach P., Lockatell V. (2013). Interleukin-33 potentiates Bleomycin-induced lung injury. *American Journal of Respiratory Cell and Molecular Biology*.

[18] Li D., Guabiraba R., Besnard A.-G. (2014). IL-33 promotes ST2-dependent lung fibrosis by the induction of alternatively activated macrophages and innate lymphoid cells in mice. *Journal of Allergy and Clinical Immunology*.

[19] Moussion C., Ortega N., Girard J.-P. (2008). The IL-1-like cytokine IL-33 is constitutively expressed in the nucleus of endothelial cells and epithelial cells in vivo: a novel ‘Alarmin’?. *PLoS ONE*.

[20] Pichery M., Mirey E., Mercier P. (2012). Endogenous IL-33 is highly expressed in mouse epithelial barrier tissues, lymphoid organs, brain, embryos, and inflamed tissues: in situ analysis using a novel Il-33-LacZ gene trap reporter strain. *Journal of Immunology*.

[21] Ohno T., Oboki K., Kajiwara N. (2009). Caspase-1, caspase-8, and calpain are dispensable for IL-33 release by macrophages. *Journal of Immunology*.

[22] Préfontaine D., Lajoie-Kadoch S., Foley S. (2009). Increased expression of IL-33 in severe asthma: evidence of expression by airway smooth muscle cells. *Journal of Immunology*.

[23] Kopach P., Lockatell V., Pickering E. M. (2014). IFN-*γ* directly controls IL-33 protein level through a STAT1- and LMP2-dependent mechanism. *The Journal of Biological Chemistry*.

[24] Bruce A. G., Linsley P. S., Rose T. M. (1992). Oncostatin M. *Progress in Growth Factor Research*.

[25] Miyajima A., Kinoshita T., Tanaka M., Kamiya A., Mukouyama Y., Hara T. (2000). Role of oncostatin M in hematopoiesis and liver development. *Cytokine and Growth Factor Reviews*.

[26] Richards C. D. (2013). The enigmatic cytokine oncostatin m and roles in disease. *ISRN Inflammation*.

[27] Hermanns H. M. (2015). Oncostatin M and interleukin-31: cytokines, receptors, signal transduction and physiology. *Cytokine and Growth Factor Reviews*.

[28] Simpson J. L., Baines K. J., Boyle M. J., Scott R. J., Gibson P. G. (2009). Oncostatin M (OSM) is increased in asthma with incompletely reversible airflow obstruction. *Experimental Lung Research*.

[29] Mozaffarian A., Brewer A. W., Trueblood E. S. (2008). Mechanisms of oncostatin M-induced pulmonary inflammation and fibrosis. *Journal of Immunology*.

[30] Fritz D. K., Kerr C., Fattouh R. (2011). A mouse model of airway disease: oncostatin M-induced pulmonary eosinophilia, goblet cell hyperplasia, and airway hyperresponsiveness are STAT6 dependent, and interstitial pulmonary fibrosis is STAT6 independent. *Journal of Immunology*.

[31] Wong S., Botelho F. M., Rodrigues R. M., Richards C. D. (2014). Oncostatin M overexpression induces matrix deposition, STAT3 activation, and SMAD1 dysregulation in lungs of fibrosis-resistant BALB/c mice. *Laboratory Investigation*.

[32] Faffe D. S., Flynt L., Mellema M. (2005). Oncostatin M causes eotaxin-1 release from airway smooth muscle: synergy with IL-4 and IL-13. *Journal of Allergy and Clinical Immunology*.

[33] Arshad M. I., Guihard P., Danger Y. (2015). Oncostatin M induces IL-33 expression in liver endothelial cells in mice and expands ST2^+^CD4^+^ lymphocytes. *American Journal of Physiology—Gastrointestinal and Liver Physiology*.

[34] Sundnes O., Pietka W., Loos T. (2015). Epidermal expression and regulation of interleukin-33 during homeostasis and inflammation: strong species differences. *Journal of Investigative Dermatology*.

[35] O'Donoghue R. J. J., Knight D. A., Richards C. D. (2012). Genetic partitioning of interleukin-6 signalling in mice dissociates Stat3 from Smad3-mediated lung fibrosis. *EMBO Molecular Medicine*.

[36] Saleh H., Eeles D., Hodge J. M. (2011). Interleukin-33, a target of parathyroid hormone and oncostatin m, increases osteoblastic matrix mineral deposition and inhibits osteoclast formation in vitro. *Endocrinology*.

[37] Rani R., Smulian A. G., Greaves D. R., Hogan S. P., Herbert D. R. (2011). TGF-*β* limits IL-33 production and promotes the resolution of colitis through regulation of macrophage function. *European Journal of Immunology*.

[38] Lefrançais E., Roga S., Gautier V. (2012). IL-33 is processed into mature bioactive forms by neutrophil elastase and cathepsin G. *Proceedings of the National Academy of Sciences of the United States of America*.

[39] Lüthi A. U., Cullen S. P., McNeela E. A. (2009). Suppression of interleukin-33 bioactivity through proteolysis by apoptotic caspases. *Immunity*.

[40] Le Goffic R., Arshad M. I., Rauch M. (2011). Infection with influenza virus induces IL-33 in murine lungs. *American Journal of Respiratory Cell and Molecular Biology*.

[41] Kearley J., Silver J. S., Sanden C. (2015). Cigarette smoke silences innate lymphoid cell function and facilitates an exacerbated type I interleukin-33-dependent response to infection. *Immunity*.

