# Supplementary material for: Regulation of IL-33 by Oncostatin M in Mouse Lung Epithelial Cells
Source: Mediators Inflamm. 2016 Sep 15;2016:9858374. doi: 10.1155/2016/9858374 (PMC5040793; doi:10.1155/2016/9858374)
Supplement: Supplementary file 1 — Supplemental Figure 1 demonstrates an induction of full-length IL-33 protein (35 kDa) and phospho-STAT3 (79, 86 kDa) by Western blot, in whole lung homogenates of C57Bl/6 mice treated with AdOSM, in comparison to AdDel70 control vector. Samples were also probed for β-Actin and total-STAT3 as loading controls. Supplemental Figure 2 shows that the antibody in Western blots detected prominent bands corresponding to full length IL-33 (approximately 35kDa) induced by OSM whereas mature IL-33 signal (recombinant mature IL-33 at approximately 18-19 kDa) was absent/low in mouse lung extracts of both AdDel70 and AdOSM-treated BALB/c mice, as well as C10 cell culture lysates stimulated with OSM. Recombinant mature IL-33 (R&D) was run on the same gel for comparison and samples were probed for β-Actin as a loading control. Supplemental Figure 3 contains results from IHC staining for IL-33 in C10 cell cultures in vitro. The data showed detectable IL-33 in nuclei of un-stimulated cells and a marked increase in nuclear staining upon OSM stimulation. Supplemental Figure 4 confirms that AdOSM treatment resulted in the same effects in BALB/c mouse lung as previously described in this system [31], including inflammatory cell accumulation in broncho-alveolar lavage (BAL) fluid and histopathology of lung tissue sections. The results indicated expected elevation of cells in the BAL fluid as well as increases in epithelial cell hyperplasia, thicker alveolar walls, and inflammatory cell accumulation in tissue sections, in response to AdOSM. [file 9858374.f1.pdf]

Supplemental figure 1

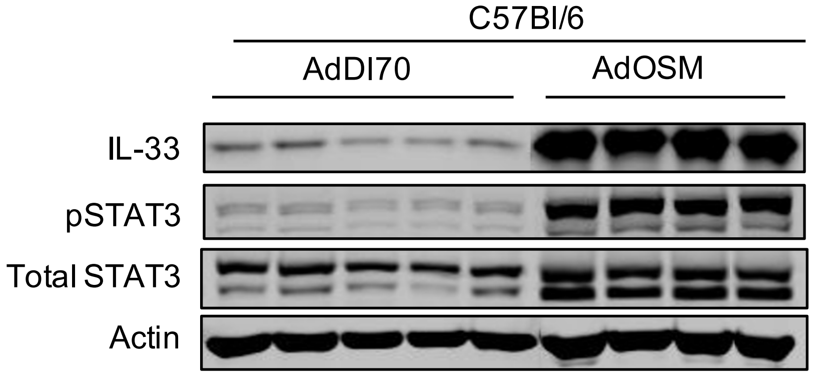

AdOSM induction of IL-33 and phospho-STAT3(pSTAT3) in C57Bl/6 mice. C57Bl/6 mice were culled after 7 days of an endotracheal administration of AdDl70 (n=5) or AdOSM (n=4) vectors and lung tissue was prepared for protein analysis as described in Methods. Lung tissue homogenate protein samples were probed for IL-33, pSTAT3, total STAT3, and β-actin as indicated.

Supplemental figure 2

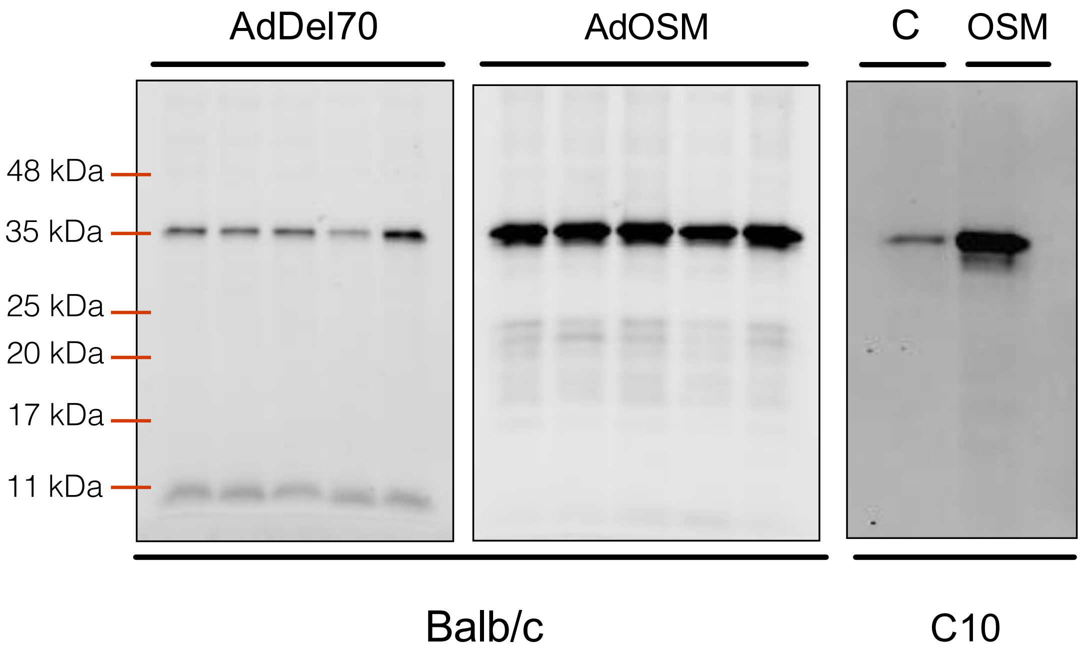

Immunoblot analysis detects primarily full length pro-IL-33. Lung tissue homogenate protein from Balb/C mice treated with AdDel70 or AdOSM vector (left and middle panel), or C10 alveolar epithelial cell protein lysates (right panel) from unstimulated (C) or OSM-stimulated cells (treated with 5 ng/ml OSM for 24 hours) were probed for IL-33 following electrophoretic separation of protein on a 12% SDS-PAGE gel.

Supplemental figure 3

IHC of C10 AEC in culture 24hr

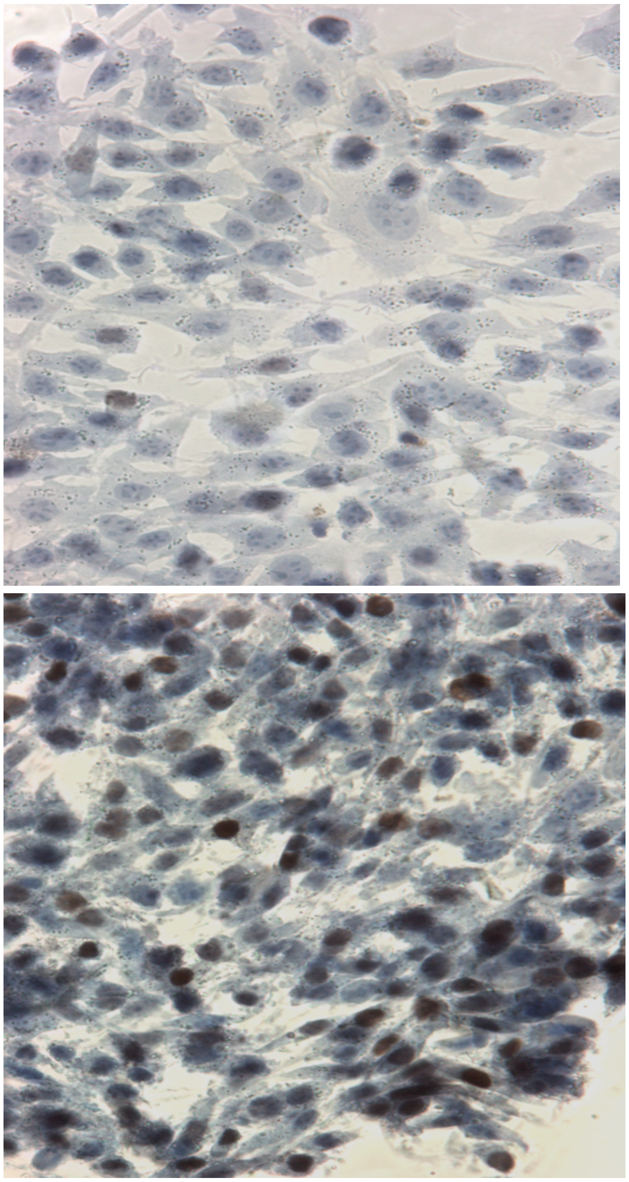

- OSM

+ OSM (10ng/ml)

OSM stimulation of C10 cells in vitro increases nuclear staining of IL-33. C10 alveolar epithelial cells (AEC) were left unstimulated (-OSM) or stimulated with OSM (10ng/ml) for 24 hours, fixed and stained for IL-33 by immunohistochemistry, as described in Methods.

Supplemental Figure 4

A

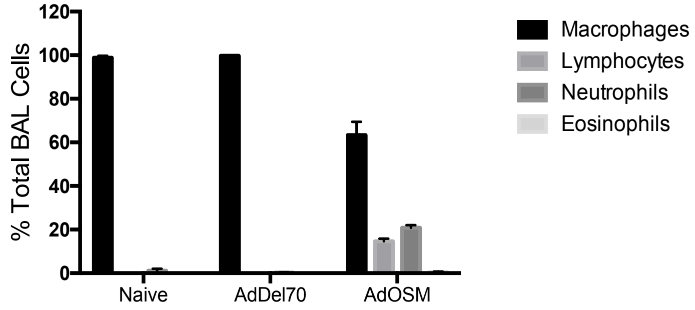

B

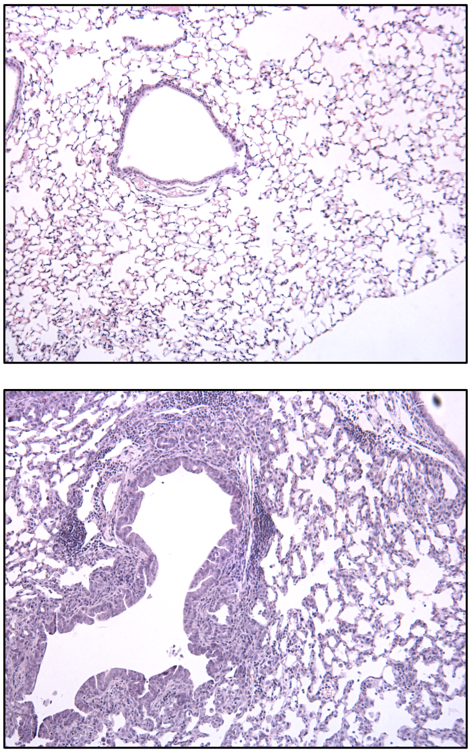

AdDel70

AdOSM

AdOSM induces cell accumulation and pathology in BALB/c mouse lung. A) Balb/C mice were treated with AdDel70 or AdOSM vector and culled after 7 days (n=3, +/- SEM) A) BAL fluid cells were analysed by differential counting as previous [31] and showed increased percentage of neutrophils and lymphocytes as previously published in BALB/c [31]. (B) Representative images of histological sections stained with H&E are shown from AdDel70 or AdOSM treated BALB/c mice as indicated (50x power).
